# The Impact of Secondhand Smoke Exposure on Children with Cystic Fibrosis: A Review

**DOI:** 10.3390/ijerph13101003

**Published:** 2016-10-12

**Authors:** Benjamin T. Kopp, Juan Antonio Ortega-García, S. Christy Sadreameli, Jack Wellmerling, Estelle Cormet-Boyaka, Rohan Thompson, Sharon McGrath-Morrow, Judith A. Groner

**Affiliations:** 1 Section of Pediatric Pulmonology, Nationwide Children’s Hospital, Columbus, OH 43205, USA; rohan.thompson@nationwidechildrens.org; 2 Center for Microbial Pathogenesis, The Research Institute at Nationwide Children’s Hospital, Columbus, OH 43205, USA; 3 Paediatric Environmental Health Specialty Unit, Department of Pediatrics, Clinical University Hospital Virgen of Arrixaca, Murcia 30120, Spain; ortega@pehsu.org; 4 Eudowood Division of Pediatric Respiratory Sciences, Johns Hopkins School of Medicine, Baltimore, MD 20205, USA; ssadrea1@jhmi.edu (S.C.S.); smcgrath@jhmi.edu (S.M.-M.); 5 Department of Veterinary Biosciences, The Ohio State University, Columbus, OH 43210, USA; wellmerling.2@osu.edu (J.W.); Estelle.Cormet-Boyaka@cvm.osu.edu (E.C.-B.); 6 Section of Ambulatory Pediatrics, Nationwide Children’s Hospital, Columbus, OH 43205, USA; judith.groner@nationwidechildrens.org

**Keywords:** tobacco, cystic fibrosis, pediatric

## Abstract

Secondhand smoke exposure (SHSe) has multiple adverse effects on lung function and growth, nutrition, and immune function in children; it is increasingly being recognized as an important modifier of disease severity for children with chronic diseases such as cystic fibrosis (CF). This review examines what is known regarding the prevalence of SHSe in CF, with the majority of reviewed studies utilizing parental-reporting of SHSe without an objective biomarker of exposure. A wide range of SHSe is reported in children with CF, but under-reporting is common in studies involving both reported and measured SHSe. Additionally, the impact of SHSe on respiratory and nutritional health is discussed, with potential decreases in long-term lung function, linear growth, and weight gain noted in CF children with SHSe. Immunologic function in children with CF and SHSe remains unknown. The impact of SHSe on cystic fibrosis transmembrane conductance regulator (CFTR) function is also examined, as reduced CFTR function may be a pathophysiologic consequence of SHSe in CF and could modulate therapeutic interventions. Finally, potential interventions for ongoing SHSe are delineated along with recommended future areas of study.

## 1. Introduction

Cystic fibrosis (CF) is a systemic disorder caused by genetic mutations in the cystic fibrosis transmembrane conductance regulator (CFTR) channel that results in dysfunctional chloride ion transport. Patients with CF characteristically suffer from persistent sinopulmonary infections, pancreatic malabsorption, systemic inflammation, and a progressive decline in pulmonary function leading to a shortened lifespan. Disease progression in CF is varied among patients and can be impacted by both genetic and environmental modifiers [1]. One important environmental modifier in other chronic lung disorders is secondhand smoke exposure (SHSe) [2,3]. SHSe can disrupt normal lung development as well as overall respiratory health in children [2]. Tobacco smoke arises as a mixture of the exhaled smoke directly from the smoker as well as sidestream smoke from the burning cigarette itself. Additionally, tobacco smoke components can deposit on indoor surfaces and cause further damage as thirdhand smoke through chemical reactions or re-entry into the indoor air environment [4]. Electronic cigarette vapor represents an emerging field of interest in regards to children’s respiratory health, but electronic cigarettes have not been studied to date in CF. Research to date in CF has focused mostly on combustible sources of SHSe such as cigarettes, and has not differentiated thirdhand smoke or included electronic cigarette vapor, and thus this review will focus on SHSe and its interactions in CF.

Despite ongoing initiatives to limit tobacco smoking, children’s SHSe remains prevalent globally, with recent global surveys reporting up to 79% of children exposed in some countries [5]. Currently, the CF Foundation recommends that all patients with CF avoid SHSe, but in 2014, 23% of individuals with CF reported monthly or more frequent SHSe [6]. However, this was self-reported data from the U.S. registry, and likely underestimates true SHSe due to a reporting and social desirability bias [7].

Cigarette smoke is composed of over 5000 chemicals, which can be directly carcinogenic or combine to provide detrimental health risks in children [8]. Notable non-carcinogenic risks in children include increases in vascular and oxidative stress which can lead to serious long-term health consequences [9,10]. Thus, our understanding of SHSe in CF has many important health implications in a vulnerable population with chronic lung disease.

In this review, we will summarize the current literature regarding SHSe in children with CF, with the goal of identifying knowledge gaps to be addressed in future research efforts.

## 2. Methods

We utilized a systematic research strategy including original research publications and published abstracts. Studies were collated using the PubMed, Embase, and Google Scholar journal databases with removal of duplicate studies, utilizing the keywords: cystic fibrosis, smoking, smoke, passive, secondhand, nicotine, cotinine, tobacco, and cigarette. Studies were included from 1990 to 2016, with 286 studies initially identified. Animal studies were included. Abstracts were reviewed for relevance and selected manuscripts were then reviewed in full if they contained objective and/or subjective measurement of SHSe along with clinical data related to CF outcomes. Relevant articles were also reviewed from the reference lists of the collected studies.

## 3. Results

### 3.1. Identifying Secondhand Smoke Exposure in Cystic Fibrosis

The identification of SHSe in children can be accomplished through a variety of methods including direct and caregiver survey measures. A standardized pediatric SHSe survey is available through the American Academy of Pediatrics Julius B. Richmond Center of Excellence [11], but this survey has not been used in CF to date. Nicotine and its metabolites such as cotinine can be measured directly as surrogates of SHSe in a variety of biologic samples. Cotinine is the major metabolite of nicotine and can be measured in biological fluids such as blood, urine, hair, and saliva [12]. Cotinine has a longer half-life than nicotine (18–20 h vs. nicotine’s half-life of 1–2 h) [13]. Nicotine can also be measured directly in hair and toenails [12]. All of these measures except blood are relatively non-invasive and easy to obtain. Hair and toenail measures have the advantage of representing approximately one month’s SHSe and the ability to store specimens at room temperature, as opposed to measurements of SHSe in urine, blood, and saliva [12]. Therefore, hair and toenail nicotine or cotinine are better markers of long-term SHSe and less subject to day-to-day exposure variability. When biological fluids are used for measurement, cotinine is the preferred biomarker, as it reflects approximately one day’s exposure and can be obtained in young children with scarcity of hair or toenails. Objective measures of SHSe may be limited by factors such as nicotine metabolism, which can vary by gender, race, and cigarette type according to activity of the enzyme cytochrome P450 2A6 (CYP2A6) [14].

Quantitative identification of SHSe in children with CF via biomarker measurement has only been accomplished in five studies [15,16,17,18,19] (Table 1) mostly from Europe, with no children under the age of five included. Biomarker measurements of SHSe (nicotine metabolites) were reported in approximately half of the children with CF, with a range of 44%–54.8% exposed. A sixth study detected positive salivary cotinine in 33% of the mothers of infants with CF [20], but did not measure the infants directly, and therefore may not have captured all sources of the infant’s exposure. Quantitative measurement of SHSe via biomarkers resulted in 35% more children being classified as exposed compared with parental report of SHSe [17]. However, when trained professionals collected the SHSe reports, SHSe was actually 10% higher than the SHSe measured [19]. In the last study, the correlation between the grade of exposure among the patients via the questionnaire and their urinary cotinine levels was strong (0.77) [19]. All six studies utilized urine or salivary measurements of cotinine [15,16,17,18,19,20], and no studies used long-term measures of exposure such as hair nicotine or cotinine [21].

Studies with parental-reported SHSe have a wider range of exposure rates than the quantified studies: between 10% and 76% of children and infants with CF had parental-reported SHSe (Table 2) [22,23,24,25,26,27,28,29,30,31,32,33,34,35,36,37,38]. However, the parental-reported studies included subjects from a wider range of countries including the United States, and included a larger registry and cohort studies with more subjects available than in the quantified SHSe studies. SHSe survey reporting methods differ from study to study and are not standardized among CF centers collecting data for national registries. Despite the limitations of parental-reported SHSe, these data are important as they are the only data available in the largest and most diverse study populations; without quantitative measurement, these rates most likely underestimate actual SHSe.

There are three studies of infants with CF from the United States, Australia, and Europe. Parental reported SHSe ranged from 26.3% to 44% in these countries [22,30,33]. Early SHSe is critically important as it impairs early lung development in non-CF populations [39]. Infants may be at the highest risk of SHSe due to a greater percentage of times indoors, higher respiratory rates, and more hand-oral behaviors than older children [2]. Children may also be exposed from a variety of sources, including parents and other household contacts, or through close contacts, such as grandparents or childcare arrangements. Nicotine is deposited on household surfaces and can therefore be re-aerosolized and inhaled or ingested by young children via touching of surfaces prior to contact with oral mucous membranes. However, it is unknown if the disproportionate effects of SHSe in young children exist in CF without objective measurements of exposure. The differences between quantified and parental-reported SHSe highlight the limitations of parental reporting in identifying SHSe in children with CF due to underreporting, the lack of information on objective exposure in young children with CF, and limited objective studies on children outside of Europe.

### 3.2. Secondhand Smoke Exposure and Cystic Fibrosis Respiratory Health

Both in utero and postnatal SHSe have been associated with reduced lung function in children without CF [2]. However, the impact of SHSe on respiratory health in children with CF has been unclear to date. Large, retrospective studies such as the United States CF Twin and Sibling study demonstrated a decrease in the forced expiratory volume in one second (FEV_1_) in children with SHSe [28]. This effect was amplified by the presence of variations in the *transforming growth factor beta* (*TGF*β*1*) gene. The EPIC observational study also determined that maternal smoking was associated with decreased FEV_1_ at age 6–7 years, in agreement with a single center study on maternal smoking utilizing cotinine levels [19,23]. Smyth reported an associated decrease in FEV_1_ by 4% and forced vital capacity (FVC) by 3% for every 10 cigarettes smoked in the household each day [15]. These findings are in contrast to other smaller observational studies, which did not demonstrate decreased FEV_1_ in children with CF and SHSe [26,40]. These differences may be in part due to the smaller cohort numbers in the negative studies. Recently our group demonstrated that infants with CF and current SHSe have increased bronchodilator responsiveness and air trapping on infant pulmonary function testing, but no differences in FEV_1_ compared with infants who do not have SHSe were found [22]. Information on in utero SHSe was not available in this study and may also contribute to airway dysfunction. These findings suggest that SHS-associated differences in large airway function may not manifest until later in life, but other signs of airway dysfunction are present in infancy. Alternatively, FEV_1_ may be a less sensitive measure of response to SHSe in young children with CF, as other lung function measures, such as impulse oscillometry, may be more sensitive than FEV_1_ in identifying airflow obstruction in young children with CF [41].

Although not consistent across all studies, one group demonstrated wide-ranging pulmonary abnormalities associated with SHSe including a five-fold increase in the number of pulmonary-related hospitalizations during the previous year and decreased Shwachman scores (which account for pulmonary exam, activity tolerance, nutritional status, and imaging findings) [36].

As many end-stage patients with CF require lung transplantation, it is important to note that there is a significant decrease in survival for CF patients who receive lungs from a donor with a history of smoking [42]. While transplanted lungs do not directly reflect SHSe, they represent another indicator of the direct toxicity of cigarette smoking to lung tissue and function. Despite these studies, data on SHSe and lung function in CF with and without transplant has been biased by a lack of standardized exposure questions, reporting preferences, and a lack of objective biomarker quantification of SHSe.

Finally, there have been no published studies to date on the impact of SHSe on lung growth in CF or pulmonary immunologic responses. Parental-reported SHSe was associated with increases in methicillin-resistant Staphylococcus aureus and anaerobic bacterial growth in oropharyngeal cultures from infants with CF in the first year of life [22]. SHSe also impairs bacterial phagocytosis in a CFTR-dependent manner, further suggesting a direct impact on bacterial clearance in CF [43]. Therefore, CF patients may be uniquely vulnerable to the immunologic impairments of SHSe, warranting further research in this area in the future.

### 3.3. Secondhand Smoke Exposure and Cystic Fibrosis Nutritional Health

In addition to an impact on respiratory health, postnatal SHSe causes micronutrient deficiencies [44], decreases food intake [45], and may stunt growth in children without CF [46]. Nutritional health in CF in the context of SHSe has mostly been examined in retrospective or observational studies. Only one study that objectively measured SHSe in CF examined growth, with no association between exposure and weight [15]. No other nutritional parameters were examined in that study. Based on parental reporting, SHSe has been associated with decreased linear growth in children with CF aged 6–11 years [34], as well as decreased linear growth and weight over the first year of life in infants with CF [22]. Additionally, in a unique study from 1990, when children with CF still attended summer camps together, children with SHSe demonstrated growth improvement during the period of camp attendance, possibly associated with temporary removal from their SHS-exposed environments [37]. Although a camp exposure may be associated with increased medication adherence and medical monitoring, children with SHSe gained significantly more weight during the two weeks of camp than did the children without SHSe, which suggests that a potential nutritional benefit of SHSe removal in CF can be seen almost immediately. There may be other socioeconomic or food preference differences between smoking and non-smoking households which could also contribute to these differences in weight gain. Finally, maternal prenatal smoking is associated with worse baseline nutritional status in CF, highlighting the importance of prenatal counseling in CF [29]. There are no studies on the impact of SHSe on micronutrients or food intake in CF.

### 3.4. Secondhand Smoke Exposure and Socioeconomic Status

Parental smoking is closely linked to socioeconomic status [47], thereby providing additional factors which can influence health in children with SHSe. In the U.S. Cystic Fibrosis Twin and Sibling Study SHSe was associated with lower socioeconomic status [28]. However, several subsequent analyses by the authors demonstrated that socioeconomic status did not confound the relationship between SHSe and lung function in CF. Similarly, ongoing work from the EPIC observational study demonstrated that SHSe was more prevalent in households with low socioeconomic status, but the effect of socioeconomic status on health outcomes was independent of SHSe [48]. Socioeconomic status and SHSe were both associated with decreases in lung function, growth, and increased pulmonary exacerbations.

### 3.5. Cigarette Smoke and Cystic Fibrosis Transmembrane Conductance Regulator Function

To date, studies have focused on the effect of cigarette smoke, representing first-hand smoking, on expression and function of CFTR and no studies have evaluated the effect of SHSe on CFTR. Cigarette smoke was first reported to inhibit chloride secretion in excised canine tracheas in 1983 [49]. CFTR was later identified as the channel responsible for smoke-induced inhibition of chloride transport as expression and function of CFTR in tobacco smokers was compromised [50]. It was also demonstrated that cigarette smoke could reduce the expression and function of CFTR in human bronchial and intestinal epithelial cells [50]. In the lung, CFTR is present at the plasma membrane of bronchial epithelial cells and regulates hydration of the airway surface liquid layer (ASL) [51]. In CF, absence of functional CFTR results in impaired mucociliary clearance due to reduced ASL. Accordingly, several studies reported that cigarette smoke impairs CFTR function and consequently reduces ASL [52,53].

Studies from our group and others focused on understanding the mechanism by which cigarette smoke negatively regulates CFTR. Our group identified the MEK/Erk signaling pathway, also known as the MAPK pathway, as the main pathway activated by cigarettes smoke and responsible for alteration of CFTR expression and function in primary human airway epithelial cells and the human bronchial epithelial cell line 16HBE [52]. Interestingly, CFTR suppression could be prevented by treatment with the antioxidant *N*-acetylcysteine (NAC) suggesting that nutritional supplements could modulate the effect of cigarette smoke. In addition, inhibition of the ERK pathway using pharmacological inhibitors could reverse the inhibitory effect of cigarette smoke on CFTR function and restore ASL in primary human airway epithelial cells [52]. Cigarette smoke exposure also leads to an increase in cytosolic calcium concentration in human bronchial epithelial cells [53]. The excess cytosolic calcium was found to come from the lysosomes, and not the endoplasmic reticulum or mitochondria. Both calcium chelation with 1,2-Bis(2-aminophenoxy)ethane-*N*,*N*,*N*′,*N*′-tetraacetic acid tetrakis(acetoxymethyl ester (BAPTA-AM) and lysosomal inhibition with bafilomycin-A1 could prevent cigarette smoke-induced CFTR internalization [53]. Interestingly, elevated calcium concentration has been shown to activate the MEK/ERK signaling pathway. Whether ERK activation by cigarette smoke occurs downstream of intracellular calcium release or not requires additional studies.

When cigarette smoke enters the lung, it also interacts with resident and circulating immune cells. Multiple intercellular pathways likely link cigarette smoke exposure and CFTR dysfunction. One particularly interesting enzyme is neutrophil elastase (NE). Secreted by neutrophils and macrophages, NE is a serine protease that hydrolyzes many different proteins including collagen, elastin, and various bacterial proteins [54]. A 2013 study found that NE degraded CFTR in NCI-H292 cells and in mice through activation of calpains, a class of non-lysosomal cysteine proteases which are activated by calcium, resulting in loss of CFTR function [55]. Further work is needed to elucidate the full impact of SHSe on circulating immune cells in CF.

### 3.6. Interventions for Secondhand Smoke Exposure

Interventions developed with the goal of preventing and eliminating SHSe have evolved over time through different methods. The most common implementation strategies include face-to-face and telephone cessation counseling. Telephone-based tobacco cessation programs in adults without CF have reported up to 12% reduction in smoking, with high satisfaction rates in the intervention population [56]. Telephone interventions have enhanced efficacy when used in combination with pharmacological tobacco cessation therapies resulting in cessation in up to 25% of subjects [57]. This suggests that outcomes may also improve with increased follow-up and contact. Tobacco cessation interventions for youth have shown better results in programs that incorporate elements sensitive to stage of change, motivational enhancement, and cognitive behavioral therapy, rather than pharmacological interventions [58]. Telephone counselling [59] (OR 1.37, CI 1.26–1.50), mobile phone applications [60] (OR 1.67, CI 1.46–1.90), and nursing interventions [61] (OR 1.29, CI 1.20–1.39) are effective approaches to smoking cessation, and nurses trained in these methods have been shown to improve the results of interventions. To date, only one center has published a direct interventional study in patients with CF and SHSe [18,19]. This study involved proactive telephone counseling that used active recruitment and targeted both patients and other family members living at home. The intervention included four principal messages: (a) the short- and long-term effects of smoking in CF, with an emphasis on the health benefits of quitting; (b) strategies for eliminating the patient’s tobacco use and/or exposure; (c) appropriate motivation based on which stage in the process of abandoning addictions the individual is at, according to Prochaska and DiClemente [62] (i.e., precontemplation, contemplation, preparation, action, and relapse); and (d) an offer of face-to-face tobacco cessation services and telephone support. This comprehensive counseling message and support significantly increased the likelihood of a smoke-free home for children with CF (OR 1.26, CI 1.05–1.54) [18]. The involvement and buy-in of individual parents and families of patients with CF in these initiatives is critical since they are the main focus of this type of program. To achieve successful initiation of this type of program at an individual center, it is important to have a planning meeting with stakeholders, CF staff, a tobacco cessation expert, and parents or community members.

## 4. Future Directions

Evidence exists that SHSe is a potential CF disease modifier. In order to prevent continued morbidity attributable to SHSe in CF, further studies and education on smoking cessation interventions for parents of children with CF are needed that are easily implementable on a wide scale. Raising awareness of the harmful effects of SHSe within the context of patients with CF is a strategy that is relevant, sustainable, and transferable to various world regions. Widespread implementation of published interventions for tobacco cessation and SHSe reduction [18,19] could alleviate the cost burden associated with treating the long-term impacts of tobacco-related diseases in CF. Trained cessation professionals are needed in the environmental health approach for the treatment of CF. We suggest that, in the future, tobacco prevention and cessation programs should be a measure of the quality of care in individual CF centers.

There are several other potential areas of future research involving SHSe in CF that are secondary to exposure reduction measures. First, objective measurements of SHSe need to be utilized in studies using available biomarkers to accurately quantify exposure and correctly classify children as being exposed or unexposed to smoke, especially in the youngest and most vulnerable populations. Because SHSe is likely an important disease modifier in CF, its measurement should be considered in studies with pulmonary function or respiratory symptoms as outcomes. Second, a standard approach to screening for smoke exposure that includes new exposures such as electronic cigarettes in CF and various tobacco products for teenagers needs to be adopted throughout all CF centers in order to facilitate research on a broader scale, as well as direct interventions at the clinical level. This would help in conducting longitudinal studies to look at the long-term impact of SHSe on lung function in CF. Third, studies are needed that look into the mechanisms through which SHSe impacts CF in order to elucidate new therapeutic targets. Fourth, as new CFTR modulators are becoming available to treat CF, it is important to determine the impact SHSe can have on those treatments.

## 5. Conclusions

CF remains a phenotypically diverse disease due to genetic and environmental modifiers of disease severity. While the search for a definitive cure of CF continues, it is important to identify treatable disease modifiers such as SHSe that can negatively impact all aspects of CF from lung function to nutritional outcomes.

## Figures and Tables

**Table 1:** Summary of studies with quantification of tobacco metabolites in cystic fibrosis (CF).

Authors	Study Date	Measure	SHSe Measured	SHSe Reported	#Subjects	Average Age (Years)
Smyth et al.	1994	Urine cotinine	46.0%	Not reported	57	None given Range 5.0–16.0
Kohler et al.	1999	Urine nicotine, cotinine, and trans-3′-hydroxycotinine	54.8%	19.4%	31	8.6
Smyth et al.	2001	Urine and salivary cotinine	44.0%	Not reported	34	12.0
Ranganathan et al.	2001	Salivary cotinine	33.0% (maternal only)	Not reported	33	0.6
Ortega-García et al.	2012	Urine cotinine	49.2%	59.8%	97	18.7
Ortega-García et al. Intervention study	2016	Urine cotinine	52.4% pre 25.0% post	62.0% pre 36.9% post	88	23.6

SHSe: secondhand smoke exposure.

**Table 2:** Summary of studies with SHS exposure via parental report.

Authors	Study Date	SHSe Reported	#Subjects	Average Age (Years)
Gilljam et al.	1990	68.8%	32	10.5
Rubin	1990	55.8%	43	9.1
Campbell et al.	1992	64.0%	44	9.0
Butz et al.	1992	29.1%	103	Parents only
Kovesi et al.	1993	29.8%	325	9.8–11.0
Weaver et al.	1994	26.3%	38	Infants, birth
Verma et al.	2001	76.0%	129	N/A
Beydon et al.	2002	21.1%	38	5.2
Ranganathan et al.	2003	28.0% (maternal)	47	0.58
Collaco et al.	2008	23.2%	812	16.8–19.5
Rosenfeld et al.	2010	23.9% (maternal)	1672	5.7
Collaco et al.	2011	33.8%	1313	N/A
		29.4%	8477	
Ren et al.	2012	10.0%	93	4.1
Rosenfeld et al.	2012	29.2%	866	4.6
Oates et al.	2015	24.5%	110	11.2
Sanders et al.	2015	47.5%	484	2.0
Kopp et al.	2015	44.0%	75	0.33
